# Health state utilities for infertility and subfertility

**DOI:** 10.1186/s12978-019-0706-9

**Published:** 2019-05-03

**Authors:** Marieke Krol, Annemiek Nap, Renée Michels, Christiaan Veraart, Lucas Goossens

**Affiliations:** 1 IQVIA, Herikerbergweg 314, Amsterdam, 1101 CT The Netherlands; 2 Rijnstate Hospital Arnhem, Wagnerlaan 55, Arnhem, 6815 AD The Netherlands; 3 Merck B.V, Tupolevlaan 41–61, Schiphol-Rijk, 1119 NW The Netherlands; 4 Merck KGaA, Darmstadt, Germany; 5 Erasmus University Rotterdam, Erasmus School of Health Policy & Management, Burgemeester Oudlaan 50, Rotterdam, 3062 PA The Netherlands

**Keywords:** Infertility, Subfertility, Fertility problems, Quality of life, Time trade-off, Utility

## Abstract

**Background:**

Health state utility values allow for comparison of treatments across different diseases. Utility values for fertility-impaired health states are currently unavailable. Such values are necessary in order to determine the relative costs-effectiveness of fertility treatments.

**Methods:**

This study aimed to determine utility weights for infertile and subfertile health states. In addition, it explored the Dutch general population’s opinions regarding the inclusion of infertility treatments in the Dutch health insurers’ basic benefit package. An online questionnaire was designed to determine the health-related quality of life values of six fertility-impaired health states. The study population consisted of a representative sample of the Dutch adult population. Respondents were asked to evaluate the health states through direct health valuation methods, i.e. the Visual Analogue Scale (VAS) and the Time Trade-Off (TTO) method. In addition, respondents were asked about their opinions regarding reimbursement of fertility-related treatments.

**Results:**

The respondents’ (*n* = 767) VAS scores ranged from 0.640 to 0.796. TTO utility values ranged from 0.792 to 0.868. Primary infertility and subfertility was valued lower than secondary infertility and subfertility. In total, 92% of the respondents stated that fertility treatments should be fully or partially reimbursed by the health insurance basic benefit package.

**Conclusions:**

Having fertility problems results in substantial disutilities according to the viewpoint of the Dutch general population. The results make it possible to compare the value for money of infertility treatment to that of treatments in other disease areas. There is strong support among the general population for reimbursing fertility treatments through the Dutch basic benefit package.

## Plain English summary

World-wide, about 15 % of the people experience difficulties with getting pregnant. Several treatments are available to help people who have fertility problems. These treatments are usually too expensive for people to pay for themselves. In the Netherlands, these treatments are therefore often paid for by the health insurers. Nevertheless, it is regularly questioned whether such treatments should be paid from national health care budgets, since people may not directly consider infertility to be a condition for which society should pay the treatment.

This study was conducted to determine whether the general population thinks fertility treatments should be paid for by general means. It was also investigated how much people thought their life would be impacted if they wanted to have children, but were not able to.

Several persons (767) representing the Dutch adult general population were asked about their views on having trouble conceiving. They were asked to participate via an online questionnaire.

The results of this study showed that most persons in the Netherlands are in favour of paying for fertility treatments through the mandatory national Dutch basic health insurance package. About 10% of the people thought this should not be the case. In general, the expected impact of having fertility problems was quite high. To illustrate, this impact was comparable to having migraine attacks twice a week.

## Background

Approximately 15% of reproductive-aged couples experience infertility worldwide [19]. In this paper, infertility is defined as permanently being unable to have children and subfertility as having trouble conceiving and not knowing whether potential fertility treatments will be successful. Mascarenhas et al. estimated that 48.5 million couples worldwide are unable to fulfill their desire for a child (which was defined as not being able to conceive in the past five years). Of these, 19.2 million couples fail to have a first child and 29.3 million fail to have an additional child [15]. About half of the couples in industrialized countries facing infertility seek medical help [18]. Fertility problems affect individuals in high income countries, as well as individuals in middle- and low-income countries [15].

Despite the high number of people having fertility problems, it is regularly questioned whether this justifies a claim on national health care budgets. The difficulty is that, although fertility is seen as a normal bodily function, policy makers may not directly consider infertility to be a disease or condition to which national health care spending should be allocated. In the Netherlands, for instance, there is an ongoing debate addresses whether fertility treatments should be (fully) reimbursed (e.g. [17]). Currently, in the Netherlands, couples get a maximum of three in vitro fertilization (IVF) or intracytoplasmic sperm injection (ICSI) attempts reimbursed through the basic benefit package of the mandatory health insurance [22]. Similarly, in many other countries there is limited access to fertility care through health insurance schemes or National Health Service systems.

An important reason why policy makers limit access to fertility treatment is the pressure on health care budgets. Because budgets are limited, decisions between reimbursement of various treatments must be made. Health economic evaluations in the form of cost-effectiveness studies play an increasing role in such health care decision making. Cost-effectiveness studies inform decision makers about the relative value-for-money that treatments offer. Such studies influence whether treatments are included in the health insurance benefit packages or the national health services systems; i.e. whether patients must pay for treatment themselves or whether the costs of treatment will be reimbursed. This also applies to reimbursement decisions concerning medical help for fertility problems.

Outcomes of cost-effectiveness analysis are preferably expressed in costs per quality -adjusted life year (QALY). QALYs allow comparison of treatments and outcomes across diseases. Decision makers can for instance, compare the cost-effectiveness of fertility treatment with the cost-effectiveness of treatments for rheumatoid arthritis. To be able to compute QALY outcomes, preference-based health-related quality of life values (also called utilities) need to be available so that costs per QALY can be calculated. A recent review [12] showed that utilities for fertility-impaired health states are currently lacking. Consequently, health economic studies on fertility treatment often examine the costs of fertility treatment per live birth, rather than the costs per QALY gained. The difficulty for policy makers is that costs per live birth cannot be compared with cost-effectiveness outcomes of other medical interventions treating other diseases.

## Methods

### Aims

The objective of this study was to determine utility weights for infertile and subfertile health states by direct utility measurement in the Dutch adult population. In addition, the study explored the general populations’ views on reimbursement of fertility treatment.

### Respondents

Data were gathered in January and February 2018. A sample of the general Dutch population was obtained through an online market research company (Survey Sampling International). The sample was representative for the Dutch population (> 18 years) in terms of age and sex.

Respondents were excluded from further analysis if they spent an unrealistically short time used to answer the questions (defined as <one-third of the median completion time), if they answered a validation question and a subsequent validation question incorrectly, or if they showed other clear signs of not having answered the questions seriously, e.g. making strange remarks in open answer field.

Respondents participated anonymously and with informed consent, and all respondents agreed that their answers would be used for scientific publication. After finalization of the online questionnaire, they were rewarded with a small amount of money to be donated to a charity of their choice or the chance to win a prize for themselves.

### Sample size calculation

The study was powered based on the desired amount of uncertainty around the estimates. Given the utility scale of 0 to 1 (where 1 is considered equal to full health and 0 is considered equal to dead) a 95%-confidence interval width of 0.06 was considered acceptable. Under a normal distribution this corresponds with a standard error of 0.0153. The standard deviation was assumed to be 0.39, which is the highest standard deviation of the health state valuations in two comparable studies [11, 14].

These considerations led to a required sample size of at least 650 respondents. Based on previous experience with studies using a similar online sample and the time trade-off technique, it was expected that 95% of the responses could be used in the analyses (e.g. [1, 13]). For extra certainty a total of 750 respondents were recruited.

### Questionnaire

The questionnaire consisted of three sections: i) a background section, ii) a health state valuations section and iii) a reimbursement opinions section. The questionnaire was developed with help of a gynecologist specialized in subfertility. This specialist provided input for al fertility- related questions and helped to design realistic health state descriptions.

### Health state descriptions

Six fertility-related health states were described for which utility values were elicited. Additionally, one health state without fertility problems was described. Patients being in the fertility-impaired health states are either 1) infertile (permanently unable to have children) or 2) subfertile (having trouble conceiving and uncertain whether a fertility treatment will be successful. Furthermore, they either already have one or three children (secondary subfertility/infertility) or they do not (primary subfertility/infertility). All seven health states relate to a person who is 38 years old with an active child-wish.

**Fig. 1:**
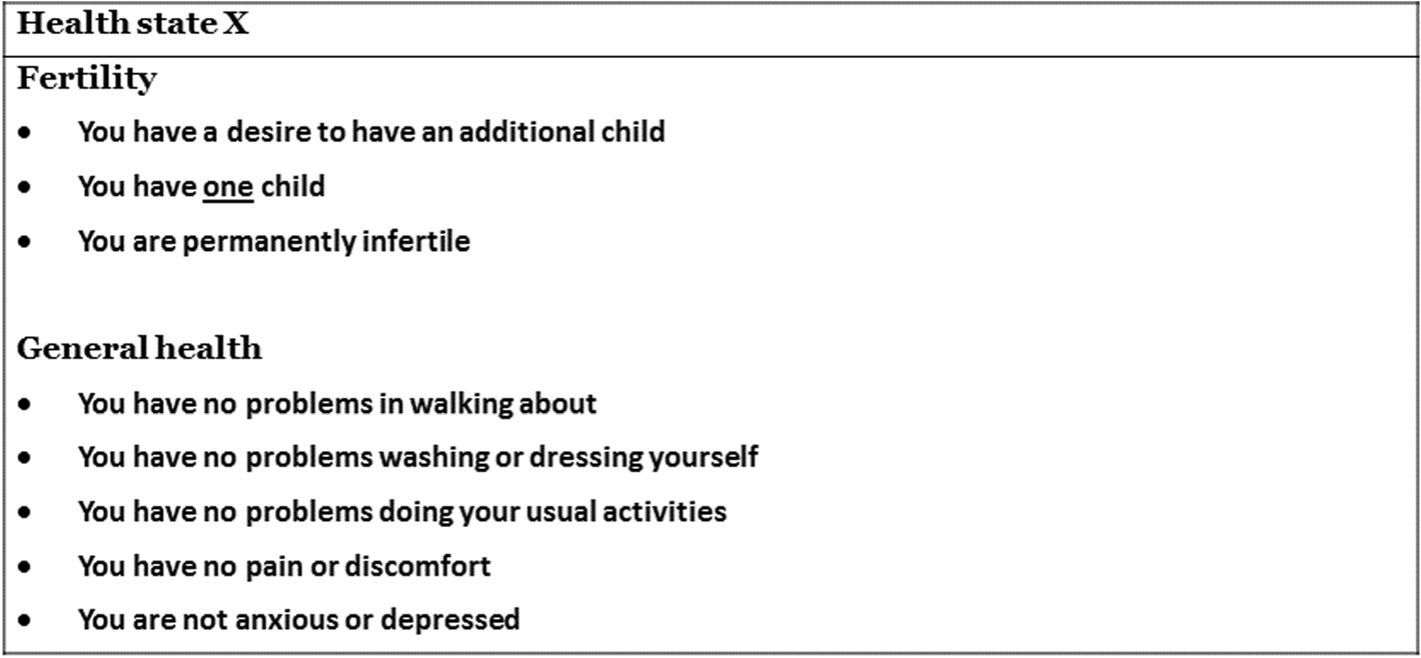
Example infertile health state description (infertile 2)

Health states definitions consisted of a general health description, based on the EuroQol 5 Dimensions, 5 level (EQ-5D-5 L) descriptive system, and a fertility-related part. An overview of all seven health states is presented in Table 1 and an example of a health state description is provided in Fig. 1.

**Table 1:** health states

	General health state	Infertile 1	Infertile 2	Infertile 3	Subfertile 1	Subfertile 2	Subfertile 3
*Fertility*							
Desire to have (more) children	NS	Yes	Yes	Yes	Yes	Yes	Yes
Current number of children	NS	0	1	3	0	1	0
Current treatment	NS	No	No	No	IVF	IVF	IVF
*General health*							
Mobility	No problems	No problems	No problems	No problems	No problems	No problems	No problems
Self-care	No problems	No problems	No problems	No problems	No problems	No problems	No problems
Daily activities	Slight problems	No problems	No problems	No problems	Slight problems	Slight problems	No problems
Pain or discomfort	Slight problems	No problems	No problems	No problems	Slight problems	Slight problems	No problems
Anxiety or depression	Slight problems	No problems	No problems	No problems	Slight problems	Slight problems	No problems
*Valuation method applied*							
VAS	+	+	+	+	+	+	+
TTO	+	+	+	+	–	–	–

*IVF* in vitro fertilization, *NS* is not specified, *TTO* time trade-off, *VAS* visual analogue scale

### Health state valuations

In economic evaluations of healthcare, health gains are expressed as QALYs. QALYs are the product of time and quality of life (expressed as utility). The latter is expressed on a scale with value 1 for perfect health and 0 for death.

In this study, two direct health state valuation methods were applied to elicit the utility of the seven health states; the Time Trade-Off (TTO) method and the Visual Analogue Scale (VAS). Both methods are widely applied and involve some biases (see for instance [4, 7]). In general, economists prefer choice based methods such as the TTO over a VAS [10].

### TTO valuations

Respondents in the TTO valuation method were asked to trade-off better health against a longer life. In this case, they chose between spending the rest of their normal remaining life expectancy of 45 years in a state with fertility problems, or living in full health for a shorter amount of time (*x* years*)*. If they were indifferent between the two options, this would mean that *x* years with perfect quality of life were equally valuable to them as 45 years in the other health state.

Mathematically, this can be expressed as follows, for health states that are considered better than death:

$$ {1}^{\ast }\ \mathrm{x}={\mathrm{U}}^{\ast }\ 45,\mathrm{or}\ \mathrm{U}=\mathrm{x}/45, $$

where U denotes the quality of life in a specific health state.

**Fig. 2:**
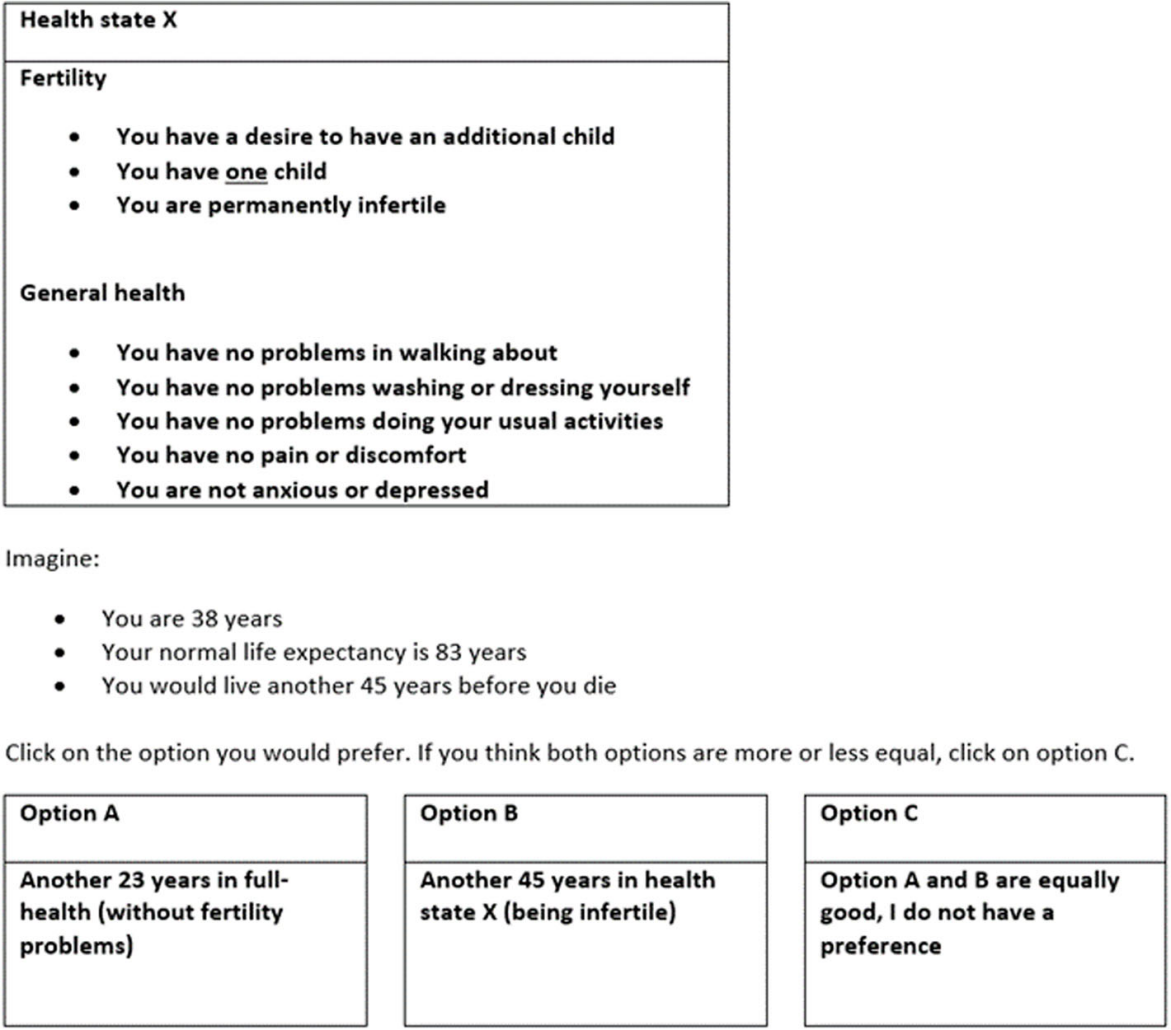
TTO Example

The indifference point *x* was determined through an iterative process in which x was varied until the respondents were indifferent. An example of the TTO valuation question is provided in Fig. 2.

The iterative process was applied as follows. In the example, respondents were first asked whether they preferred 45 years in full health (without fertility problems) or 45 years in health state X (being infertile). If the respondent chose 45 years in full health, the years in full health were cut in half. Respondents were then asked if they preferred 23 years in full health or 45 years in health state X. If they chose 45 years in health state X, the years of full health were changed to the mid-point of 23 and 45 (= 34). If in the next question they chose 45 years in health state X, years in full health were changed to the mid-point between 34 and 45 (= ~ 40). This process was repeated until either option C was chosen (i.e. point of indifference is reached) or when the point was reached that respondents were offered a choice between 44 years in full-health and 45 years in health state X. In the latter case the indifference point was assumed to be at 44.5 years in full-health (i.e. 6 months of living were assumed to be traded-off). If respondents chose option C at a certain moment, then the offer of full-health presented at that moment is the indifference point. For instance, if a respondent in Fig. 2 chose option C, the indifference point was 23 years (i.e. 22 years were traded-off). If respondents in every iteration chose to prefer the lower number of years in full-health (which was halved every question), the iteration process stopped when the years of full-health offered reached 1. Respondents were then presented with a slider between 0 and 1 to indicate how much time in full health between 0 and 1 year they felt was equal to 45 years in the impaired health state. If, for instance, they indicated 0.5 years, this meant that they had traded-off 44.5 years. The utility values of the presented health states were calculated by dividing the number of years traded-off by 45 (the normal remaining life span).

Since a TTO exercise is a relatively complex task, respondents were first asked a validation question. In the validation question a general health state (see Table 1) was presented. Respondents were asked whether they preferred 45 years in the general health state 1 (with health problems), 45 years in full-health or whether they were indifferent. If respondents stated that they preferred 45 years in health state 1 or that they were indifferent, they were subsequently asked whether they were sure in the validation question. If they answered this validation question confirmative, the respondents’ answers were excluded from further analysis.

The question was also used to check the average valuation of this health state by the sample against the valuation of the health state in the Dutch EQ-5D-5 L tariff, which is 0.778 [20].

Note that the most commonly applied time horizon in TTO questions is ten years [3]. However, as seen in Fig. 2, in this study remaining life expectancy was used instead. The principal reason for this is that if life ends after ten years, a potential child would still be young. This perspective might make it seem less desirable to have children at all. The life span of 83 years applied in this study was based on the life expectancy for a 38-year-old person in the Netherlands [5].

TTO could not be applied for the subfertile health states, since the method requires that the imperfect health state is clearly defined and described in sufficient detail. This was problematic, since the uncertainty about being able to conceive in the subfertile health states may be an important aspect influencing quality of life (i.e. the utility values). It would therefore be inconsistent to tell respondents whether infertility treatment would be successful. Moreover, it would not be realistic to remain uncertain of the IVF outcome over the entire remaining life span of 45 years of a 38-year-old. For this reason, TTO was only applied for the infertility health states and the general health description.

### Visual analogue scale valuations

**Fig. 3:**
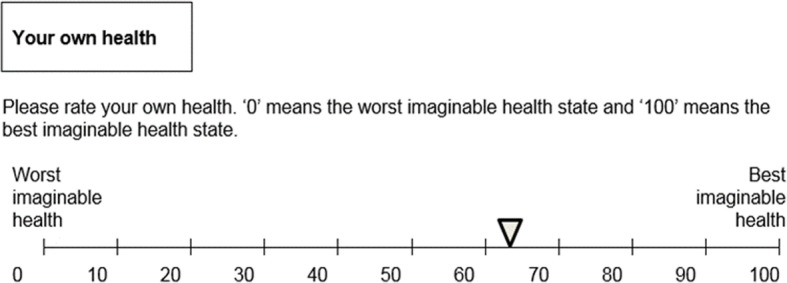
Example of Visual analogue scale

The Visual Analogue Scale (VAS) is a straightforward direct valuation method in which participants rate their own health or a described health state on a scale from 0 to 100, see for example Fig.3. This method can also be used for health states that involve uncertainty. On the VAS rating scale ‘0’ represents the worst imaginable health state and ‘100’ the best imaginable health state. Respondents were first asked to rate their own health, next they were asked to rate an impaired health state not specifically entailing fertility problems (health state 1 in Table 1) and, subsequently the infertile and subfertile health states were presented. Respondents were presented with a slider they could move between a value between 0 and 100. At the start of each health state valuation task the slider was placed in the middle (at 50). The respondent could only move to the next question after having moved the slider. VAS scores were divided by 100 to make them more comparable with TTO scores.

### Reimbursement opinions

The final section of the questionnaire consisted of questions about the respondents’ opinions regarding reimbursement of fertility-related treatments by the Dutch basic benefit package (mandatory health insurance package for the total population). Respondents were asked whether they thought fertility-related treatments (more specifically, IVF treatment) should be part of the Dutch basic benefit package (fully, not at all, partly) If they answered that fertility treatments should be partly reimbursed by the basic benefit package, they were asked how many IVF attempts they thought should be reimbursed.

### Analysis

The average TTO and VAS scores were calculated per health state for the entire sample as well as for subgroups of respondents (defined by gender, religion, age, education, experience with fertility problems, and wishing to have (more) children).

An extra step was taken to make the VAS estimates for the subfertility health states comparable to the estimates for the other health states that were elicited by TTO. Since TTO values are usually structurally higher than VAS values, these VAS values were increased by the average difference between the VAS and the TTO values of the health states for which both were available.

### Discounting

Respondents tend to value years further in the future lower than more immediate ones, which could lead them to give up future life years in exchange for utility gains relatively easily. This would result in biased utility estimates, since all years are equally valuable in the QALY concept [2]. To correct this bias, the indifference points from the TTO as well as the normal life expectancy were discounted by the 1.5% per year rate that is prescribed for future health benefits by the Dutch health economic guidelines [21].

## Results

### Study population

At the interim analysis (half way through the process of the data collection) it turned out that approximately 30% of the respondents did not give a correct answer to the validation question. Therefore, the data collection was prolonged aiming to collect a total of 950 completed questionnaires instead of the originally planned 750. Finally, 994 respondents completed the questionnaire. Of those respondents, 599 answered the previously described validation question correctly (i.e. stating that they would rather live 45 years in full health than 45 years in an impaired health state). An additional 77 respondents answered the validation question correctly after asking them if they were sure about their answer. None of the respondents correctly answering the validation question the first or second time were considered speeders. Consequently, the answers of 676 respondents were included in the analyses. The characteristics of these respondents are summarized in Table 2. A majority of the sample have children, and one-third would like to have (had) (more) children. Twelve percent of the respondents have had experience with fertility problems. The average self-reported VAS score of their own health was 0.719. The sample was representative for the Dutch population (> 18 years) in terms of age and sex. However, a higher proportion of the respondents were middle and higher educated than the adult general population. There self-reported health on the VAS was lower than reported in another general population time trade-off survey [20]. Respondents above 45 years old had on average 1.6 children, which is similar to the general population in 2017 [6].

**Table 2:** Sample characteristics

	*N* = 676
Female	0.5133
Age (SD)	45.1 (16.0)
Education, low (elementary school and lowest level of secondary education)	24%
Education, middle (highest level of secondary education)	40%
Education, high (university degree, bachelor or master)	36%
Respondents with one or more children	59%
Child-wish	33%
Fertility-related problems	12%
Self-reported health, VAS (SD)	0.719

### Health state valuations

Table 3 shows the utility weights derived with the VAS and the TTO questions. The TTO utility values of the infertile health states ranged from 0.792 to 0.868. The lowest value was given for primary infertility and the highest for secondary infertility while already having three children. The confidence intervals were narrow. The same pattern is visible for the VAS scores, but the scores are lower than the TTO scores. Likewise, the adjusted VAS scores for the subfertile health states were consistently lower than the TTO scores for the infertile health states. The lowest score, 0.726, was estimated for the first subfertile health state: a childless individual during a fertility treatment with side effects and uncertainty about the (final) outcome.

**Table 3:** VAS and TTO utilities

Health state			VAS	CI		TTO	CI	
General health state	Fertility NS	Some other health problems	0.710	0.697	0.723	0.784	0.765	0.803
Infertile 1	Primary infertility	No other health problems	0.689	0.674	0.704	0.792	0.771	0.813
Infertile 2	Secondary infertility (1 child)	No other health problems	0.751	0.738	0.763	0.845	0.825	0.864
Infertile 3	Secondary infertility (3 children)	No other health problems	0.796	0.783	0.808	0.868	0.848	0.887
			VAS	CI		Adjusted VAS	CI	
Subfertile 1	Primary subfertility	Some other health problems	0.640	0.626	0.654	0.726	0.712	0.740
Subfertile 2	Secondary infertility (1 child)	Some other health problems	0.662	0.648	0.675	0.747	0.734	0.761
Subfertile 3	Primary subfertility	No other health problems	0.675	0.661	0.689	0.761	0.747	0.775

*CI* confidence interval, *TTO* time trade-off, *VAS* visual analogue scale. For more detailed descriptions of health states see Table 1.

The sample’s valuation of the general health state (without specific information about having fertility problems or not) with mild problems on three health domains (daily activities, pain/discomfort and anxiety/depression) was very close to the value in the Dutch tariff (0.784 compared to 0.778).

This general health state was valued higher than subfertile health states 1 and 2, which were the exact same health states apart from the additional fertility information. In other words, the same health state was valued differently when the cause of the general health problems was defined, namely IVF treatment and the related uncertainty of being able to fulfill the desire to have children. This utility decrement in subfertility with ‘some other health problems’ compared to the general health state with ‘some other health problems’, is an indication that the disutility of subfertility cannot be fully captured by the EQ-5D-5 L. Lastly, primary in−/subfertility was valued lower than secondary in−/subfertility.

The TTO and adjusted VAS scores for several subsets of respondents are presented in Table 4. Older respondents valued all health states higher than younger respondents. A larger difference can be seen between people with and without the wish to have (more) children. Respondents who have experienced fertility problems themselves valued infertile problems higher (less impact on quality of life) than those who did not have experience with fertility problems. Conversely, those respondents with experience with fertility problems valued subfertile states lower (more impact on quality of life) than those without experience with fertility problems. For all other subsets, scores are very close to those for the full sample.

**Table 4:** Comparisons between groups

	General health state	Infertile 1	Infertile 2	Infertile 3	Subfertile 1	Subfertile 2	Subfertile 3
*Women*	0.803	0.788	0.843	0.873	0.704	0.825	0.874
*Men*	0.764	0.796	0.847	0.862	0.798	0.849	0.889
*Religious*	0.795	0.781	0.842	0.862	0.768	0.837	0.880
*Not religious*	0.774	0.801	0.847	0.872	0.780	0.836	0.882
*Age <45*	0.747	0.745	0.797	0.822	0.751	0.814	0.862
*Age >=45*	0.821	0.841	0.894	0.915	0.799	0.859	0.901
*Low education*	0.795	0.802	0.823	0.854	0.803	0.837	0.870
*Middle*	0.786	0.802	0.855	0.885	0.773	0.834	0.879
*High*	0.774	0.776	0.847	0.857	0.757	0.839	0.891
*Experience with fertility problems*	0.816	0.811	0.868	0.883	0.737	0.828	0.876
*No experience with fertility problems*	0.778	0.788	0.842	0.865	0.782	0.840	0.884
*Child-wish*	0.750	0.708	0.783	0.822	0.741	0.814	0.867
*No child-wish*	0.806	0.838	0.878	0.892	0.792	0.850	0.889

### Reimbursement opinions

In total 29% of the respondents stated that fertility treatments should be fully reimbursed by the health insurance basic benefit package and 8% of respondents stated fertility treatments should not be reimbursed at all. Sixty-three percent of all respondents were of opinion that fertility treatments should be partly reimbursed. Those respondents thought (on average) that 4.0 IVF attempts (SD 2.5) should be reimbursed by the basic benefit package.

## Discussion

The results of this study indicate that having fertility problems results in quite substantial disutility according to the viewpoint of the Dutch general population. Our estimates of the quality of life of patients with infertility and subfertility are in the range of those for newly diagnosed early ovarian cancer [11], having migraine attacks twice a week [16] and the quality of life of children with attention deficit hyperactive disorder with mild to moderate problems [14]. Primary infertility and primary subfertility is valued lower (stronger impact on quality of life) than secondary infertility and subfertility.

Next to the impact of subfertility and infertility on quality of life, this study also investigated the view of the Dutch general population on the reimbursement of fertility related treatments. The results show that a strong majority of the general population is in favour of including these treatments in the Dutch mandatory health insurance package (basic benefit package). Less than 10% of the general population sample is of opinion that fertility treatments should not be covered at all and over a quarter of the population thinks fertility treatments should unlimitedly be reimbursed. Individuals who were of opinion that the reimbursement of fertility treatments should be limited, indicated that (on average) 4 IVF attempts should be included in the basic benefit package. Note that currently in the Netherlands 3 IVF attempts are reimbursed.

Our study has some limitations. First, we used an online sample. Given that the TTO exercise is relatively cognitively demanding, this task may have been difficult for respondents. This idea is strengthened by the substantial proportion of respondents who answered our validation questions incorrectly and had to be removed from the sample. Additionally, our respondents were higher educated than the Dutch general population. However, our validation questions did indicate that most of the respondents understood the task and took it seriously and provided a similar value for the general (not infertility-related) health state as the Dutch EQ-5D-5 L tariff [20]. In general, using online questionnaires has some advantages (such as convenience for respondents, allowing for bigger sample sizes and the non-existence of interviewer bias) and some disadvantages (for instance, lack of possibility to give additional instructions when needed and sometimes low response rates) [9]. A second limitation is that we were not able to obtain TTO values for the subfertility health states, because it was not possible to construct a realistic TTO scenario for these health states. To cope with this, we adjusted the VAS outcomes of the subfertile health states to be better comparable with the TTO values of the infertile health states. Thirdly, although we designed the health state description with a clinical expert, we did not involve patients in this process. Fourthly, a limitation of the questionnaire concerning the viewpoint of the Dutch general population on the reimbursement of fertility related treatments, is that the questions did not inquire about the *relative* importance of reimbursing fertility treatments. Although the population may be in favour of reimbursing these treatments, the question remains whether they should be reimbursed instead of other treatments. Respondents may be inclined to state that they find it important to have all possible health care interventions reimbursed. Finally, the results may not be directly generalizable to other countries.

Some issues related to economic analyses of fertility treatment remain. For instance, there is a lack of consensus if and how the value of new life should be included in these analyses and if effects of treatments on quality of life should be captured of the couple or of actively treated individuals only [8]. Therefore, it remains important to further debate on how to capture and include all relevant costs and effects in economic analyses of fertility treatments.

To our knowledge, this was the first time that the impact of primary infertility (involuntary childlessness), secondary infertility and primary and secondary subfertility is determined in terms of utility outcomes. The reliability of these estimates was increased by the fact that our sample closely agreed with the Dutch EQ-5D tariff on the valuation of a general, non-fertility-related health state.

This allows for comparing the impact of infertility and subfertility on quality of life with the impact of other diseases on quality of life. Moreover, the values identified in this study can be used in cost-effectiveness analyses investigating the relative ‘value for money’ of fertility treatments.

## Conclusion

This study identified the utility values of health states involving subfertility or infertility and indicated that subfertility and infertility have a strong negative effect on quality of life. The identified values allow comparisons across diseases. This study also showed that there is a strong support among the Dutch general population for reimbursing fertility treatments from the Dutch basic benefit package.

## Abbreviations

EQ-5D-5 L: EuroQol 5 Dimensions, 5 level
ICSI: Intracytoplasmic sperm injection
IVF: In vitro fertilization
QALY: Quality adjusted life years
TTO: Time Trade-Off
VAS: Visual Analogue Scale
